# Genetics of Inherited Retinal Diseases in Understudied Populations

**DOI:** 10.3389/fgene.2022.858556

**Published:** 2022-02-28

**Authors:** Chitra Kannabiran, Deepika Parameswarappa, Subhadra Jalali

**Affiliations:** ^1^ Kallam Anji Reddy Molecular Genetics Laboratory, Prof Brien Holden Eye Research Centre, Hyderabad, India; ^2^ L. V. Prasad Eye Institute, Hyderabad, India; ^3^ Smt Kannuri Santhamma Centre for Retina Vitreous Services, Hyderabad, India

**Keywords:** retinitis pigmentosa, retinal dystrophies, genetics, homozygosity, gene mapping, mutations, sequencing

## Abstract

Retinitis pigmentosa is one of the major forms of inherited retinal dystrophy transmitted in all Mendelian and non-Mendelian forms of inheritance. It involves the loss of retinal photoreceptor cells with severe loss of vision or blindness within the first 2 decades of life. RP occurs at a relatively high prevalence in India and is often associated with consanguinity in certain South Asian communities where this practice is customary. This review describes the studies that have been published with regard to genetics of retinitis pigmentosa in India and neighboring South Asian countries. These populations have been understudied in these aspects although to a variable degree from one country to another. Genetic studies on RP in India have been carried out with a range of methods aimed at detecting specific mutations, to screening of candidate genes or selected genomic regions, homozygosity mapping to whole genome sequencing. These efforts have led to a molecular genetic characterization of RP in Indian families. Similar studies on large extended families from Pakistan have provided insight into several novel genes underlying the pathogenesis of these diseases. The extreme degree of clinical and genetic heterogeneity of RP renders it challenging to identify the associated genes in these populations, and to translate the research output towards better management of the disease, as there are no unifying genetic features that are characteristic of any population so far.

## 1 Background

Inherited retinal diseases are a large, heterogeneous group of disorders that are grouped according to the retinal layers primarily affected, whether syndromic or non-syndromic, and by the mode of inheritance. Retinitis pigmentosa (RP) is a hereditary retinal disorder leading to severe visual impairment or blindness in the first 2 decades of life. RP involves the death of photoreceptor cells, a process that occurs progressively with loss of both rods and cones. Though classified as a rod-cone type of disease with primary loss of rods, the pattern of disease may involve loss of cones in early stages of the disease as well, in addition to loss of rods. Typically, it manifests with night blindness, loss of visual acuity, loss of visual fields which progressively leads to “tunnel vision.” The electroretinographic responses of the photoreceptors are extinguished. It is clinically very heterogeneous in its manifestation.

This review deals with genetics of RP and related diseases in South Asian populations, with particular reference to India, but also highlights studies in this topic from Pakistan, Nepal and Bangladesh. These countries are not equally represented in the literature in the field of retinal dystrophies. Among the countries mentioned, there are a much larger number of studies on families with retinal dystrophy from Pakistan. Mutations are reported in disease-associated genes in several Pakistani families, by conventional techniques of mapping and sequencing, and more recently, by next generation sequencing (NGS). For its geographic area and population, Pakistan is not underrepresented in studies of RP, despite the higher prevalence of RP and related retinal diseases as compared to Western countries. The availability and recruitment of extended, large families residing in the same town or village has conceivably facilitated genetic studies for the identification of novel genes. The relative proportion of published literature on the genetics of RP from this region, suggests that there are about twice as many publications on Pakistani families with RP as compared to India (Zafar et al., 2017).

A discussion on the genetics and other related aspects of RP in Indian populations is provided here (Table 1) as well as representative genetic studies from the Pakistani population (Table 2). Literature in this subject is very scarce from the other countries in South Asia.

**TABLE 1:** Major methods employed in genetic studies on Retinal Dystrophies in India.

Phenotype	Method used	References
ADRP, ARRP, and XLRP	Allele-specific assays for codons 345, 347 in *RHO* gene	Dikshit and Agarwal, (2001)
ARRP	Allele-specific ligation	Sundaresan et al. (2009)
ADRP, isolate RP	Sequencing	Gandra et al. (2008)
ADRP	Linkage analysis and sequencing	Saini et al. (2012)
ADRP	Linkage analysis and sequencing	Bhatia et al. (2018)
ARRP, LCA	Homozygosity mapping, Sanger sequencing	Singh et al., 2006; Singh et al., 2009; Kannabiran et al., 2012; Srilekha et al., 2015
ARRP, LCA, CRD	Homozygosity mapping, targeted NGS	Sundaramurthy et al. (2016)
ARRP	Homozygosity mapping, exome sequencing	Goyal et al. (2016)
GFS	Direct sequencing	Manayath et al. (2014)
LCA	Targeted NGS	Srikrupa et al., 2018; Sen et al., 2020
ARRP	Whole exome sequencing	Bhatia et al., 2019; Woodard et al., 2021
Best disease	Whole exome sequencing	Nguyen et al. (2018)

Phenotypes studied, and the methods used to identify mutations in Indian families are shown above.

**TABLE 2:** Summary of Major Genetic Studies on RP and related diseases in Pakistani families.

Phenotype	Methods used	Associated gene/Locus	Authors
ARRP	Homozygosity and linkage mapping, gene prediction and transcript analysis	*RP25/EYS*/6q	Khaliq et al., 1999, Collin et al., 2008
Usher Syndrome Type 1F	Linkage analysis, physical mapping and sequencing	*PCDH15*/*USH1F*	Ahmed et al. (2001)
ARRP	Linkage analysis	*RP29* (4q32-34)	Hameed et al. (2001)
ARRP	Linkage analysis and sequencing	*RP1*/8q11	Khaliq et al. (2005)
Oguchi disease	Linkage analysis and sequencing	13q34/*GRK1* (rhodopsin kinase)	Zhang, Zulfiqar, Riazuddin et al. (2005)
ARRP	Linkage and homozygosity mapping	*RP32*/*CLCC1* (1p13-21)	Zhang, Zulfiqar, Xiao et al. (2005)
	Whole exome sequencing		Li et al. (2018)
ARRP with mental retardation	Linkage analysis and sequencing of candidate genes	*CC2D2A*/4p15.33-p15/	Noor et al. (2008)
Usher Syndrome type *1J*	Linkage mapping; sequencing of candidate genes	*CIB2/USH1J* (15q22*-*23)	Riazuddin et al. (2012)
ARRP	Linkage analysis	2p22.3-p24.1	Naz et al. (2010)
ARRP	Homozygosity and linkage analysis; sequencing	*USH3/CLRN1* (Clarin 1)	Khan et al. (2011)
Usher Syndrome type 1K	Linkage mapping	*USH1K/*10p11.21-q21.1	Jaworek et al. (2012)
ARRP	Homozygosity mapping, exome sequencing	*DHX38/*chr 16	Ajmal et al. (2014)
CRD	Exome sequencing	*CNGA3*	Shaikh et al. (2015)

The Table lists several studies on retinal dystrophies in Pakistani families in which novel loci were mapped and/or identified.

## 2 Prevalence of Retinitis Pigmentosa

The prevalence of RP and related diseases has been determined in population-based studies in different regions of India. In the state of Andhra Pradesh in Southern India, a study on the frequency and causes of blindness found the prevalence of RP to be about 1 in 1,000 population, (0.1%) in both rural and urban areas combined. Blindness due to RP accounted for almost half of all blindness due to retinal diseases (Dandona et al., 2001). Population-based studies from other states in India provide similar frequencies for RP, with about 0.17% prevalence in the state of Tamil Nadu in Southern India (Sen et al., 2008) and 0.13% in a population from Central India (Nangia et al., 2012). A hospital-based study across a L. V. Prasad Eye Institute (LVPEI) network of hospitals representing primary to tertiary eye care across Southern and Eastern India, reported a prevalence of about 0.59% for RP (Parameswarappa et al., 2021). The higher frequency of RP in this report may be relevant to an eye care institutional framework which included all patients with eye diseases reporting to the hospitals in the network. Among the modes of inheritance of the disease, the occurrence of familial disease as reported varies widely between studies, possibly due to a difference in the sizes of study populations analyzed. The prevalence of familial disease is about 0.1 (10%) based on a study of over 15,000 patients with RP presenting over a 6-year period (Parameswarappa et al., 2021). In contrast, a study of seventy-eight cases covering a 6-month period, suggested the occurrence of a familial pattern (including all modes of autosomal and X-linked inheritance) in about half of the patients. Isolate cases (patients with no family history of the disease) represent close to one-half of RP patients encountered in this setting (Kar et al., 1995).

Consanguinity is a social factor that is often associated with recessive disease, and has been suggested to be correlated to the higher occurrence of RP in Southern India, due to the common custom of consanguineous marriages in this region (Nirmalan et al., 2006). Consanguinity between two spouses increases the chances of an offspring to inherit the same disease gene from each of the parents and thereby develop recessive disease. In such families, both parents are carriers of a common disease gene that is passed down from a single ancestor. Thus, the offspring is autozygous at the disease locus (Lander and Botstein, 1987). This phenomenon of autozygosity or homozygosity by descent (HBD) applies not just to the disease locus, but extends to a fraction of the entire genome. The probability that a particular genomic region or gene is autozygous in an individual is also known as the co-efficient of inbreeding (F). The value of F is 1/16 for marriage between first cousins, 1/8 for uncle and niece. Thus, the length of the genome flanking the disease gene that is autozygous in the affected offspring varies depending on the extent of relatedness of the two parents. Despite the existence of autozygosity, the association between consanguinity and recessive disease in RP may be tenuous particularly due to the low prevalence of RP itself, as well as the frequent rate of consanguinity in the South Asian populations mentioned here. In addition, the relationship between the prevalence of RP and consanguinity was found only in the case of a particular form of consanguinity-first cousin marriages.

## 3 Clinical Presentation of Retinitis Pigmentosa

The most common inherited retinal dystrophies (IRDs) seen at L. V. Prasad Eye Institute (LVPEI), a tertiary care referral center network in Southern India, are retinitis pigmentosa, LCA, juvenile retinoschisis, achromatopsia, Stargardt’s disease and Best disease. In all forms of retinal dystrophy, a predominance of males was observed among patients presenting to the clinic. About one-third of patients with RP belonged to lower socio-economic strata. A history of consanguineous marriage was found in every sixth patient with a positive family history of the disease. A majority of patients presented in the clinic in the third decade of life with severe impairment in visual acuity. Defective night vision was a predominant clinical symptom at presentation. Other symptoms found at presentation include, reduction in peripheral and central vision, color vision abnormalities and delayed dark adaptation. The retinal features noted at presentation in this population include disc pallor (76.43% of cases), attenuated arterioles (82.61% of cases), and bone spicule pigmentation (90.15% of cases). Almost all patients were found to have retinal pigment epithelial changes (Parameswarappa et al., 2021).

The common syndromes associated with RP in this patient population are Usher Syndrome followed by Lawrence Moon Bardet Biedl Syndrome. Patients with Usher syndrome (USH) Type 1 and type 2 are more common than type 3 and present in the 1st to 2nd decades of life. Defective night vision is the major manifestation at initial visit in all three types of Usher syndrome. It is important to rule out Usher syndrome in early onset RP with hearing abnormalities. Laurence-Moon-Bardet-Biedl (LMBB) syndrome is the second most common syndromic form of RP. Patients present in 1st–2nd decade of life with severe visual acuity impairment (20/200 to 20/400) to blindness (<20/400) early in life. Severe reduction in central visual acuity is due to early macular involvement and optic atrophy. Myopia is the predominant refractive error and nuclear cataract is the common form of cataract seen in LMBB with RP. A prominent retinal feature is a diffuse or widespread retinal pigment epithelial degeneration in all patients. Patients with features of early onset RP and impairment in central visual acuity need to be evaluated for LMBB syndrome. Early visual as well as systemic rehabilitation is crucial along with supportive therapy upon detection of LMBB syndrome. Though patients with IRD in India currently do not have access to specific visual implants or gene therapy, surgical intervention for associated problems like cataract, capsular phimosis, angle closure glaucoma, retinal detachment, macular pathology etc., may be required. All IRD patients need comprehensive low vision assessment and rehabilitation.

Diagnostic and management challenges in a clinical set up in India with respect to RP and other IRDs are variable clinical presentation, lack of diagnostic and imaging tools in all hospitals, difficulty in examining all the family members, lack of uniform guidelines for genetic counselling and the high cost of genetic testing. Even where testing is available, there are gaps in genetic testing or counselling for all patients due to lack of patient awareness, acceptance, and accessibility of the services.

## 4 Genetics of Retinitis Pigmentosa and Related Disorders in India

**FIGURE 1:**
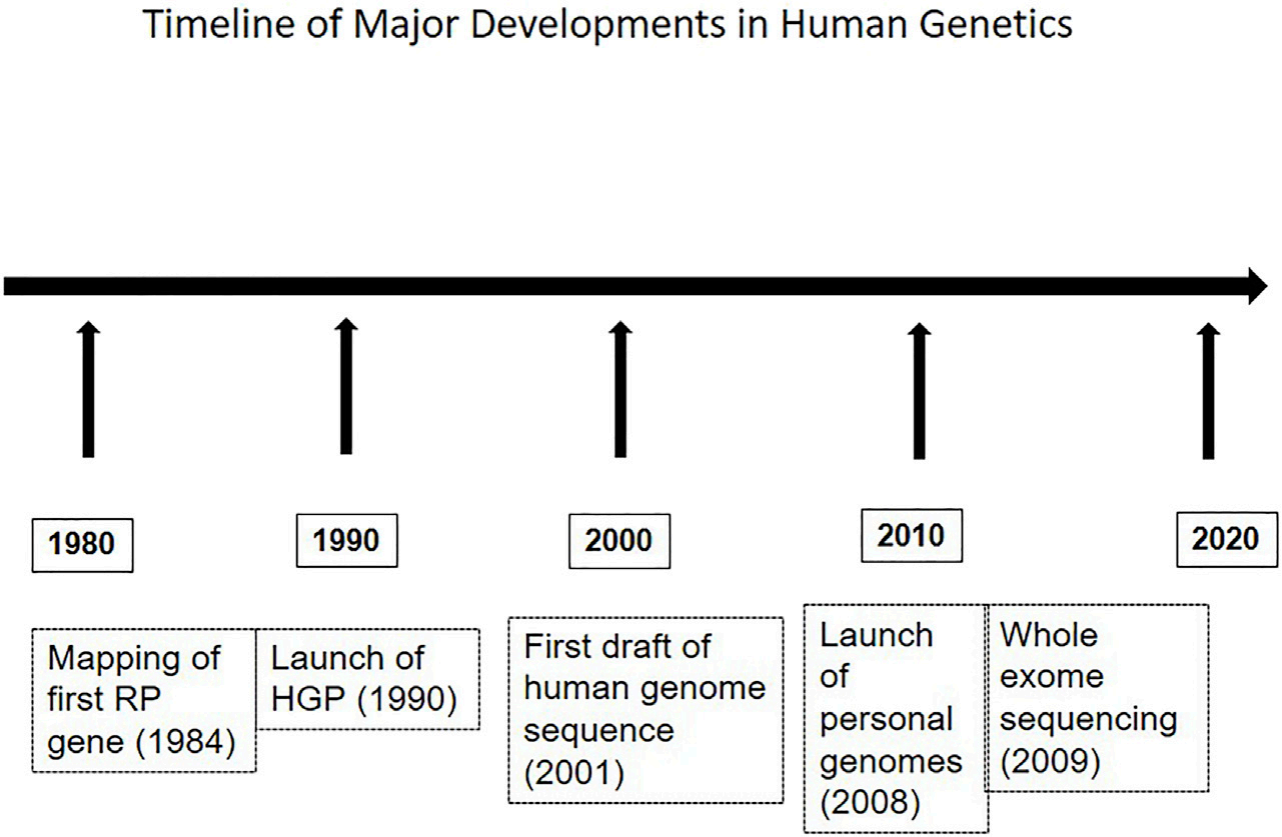
The figure depicts a sequence of major developments in genetics, relevant to the field of retinal dystrophies as well.

Molecular genetic investigations of patients and families with RP have employed a variety of methods, and these have evolved over time, in parallel with advances in the field of human genetics and genomics. Figure 1 shows a timeline of major developments in this area. The methods range from selective analysis of chosen mutations, screening of candidate genes to genome-wide approaches including mapping and exome sequencing. The methods used in various studies are summarized in Table 1. For example, allele-specific assays were used for the detection of common mutations in the rhodopsin (*RHO*) gene. These include changes at codon 345 (Val345Met and Val345leu) and codon 347 (Pro347Ser/Ala/Arg/Gln/Thr); screening of a series of about sixty-nine probands consisting of dominant, recessive, X-linked and sporadic RP, showed the codon 345 mutation in two probands. None of the patients tested had codon 347 mutations (Dikshit and Agarwal, 2001). Allele-specific assays have also been employed in a multiplexed mode for screening known mutations in RP genes. Thus, a missense change in *RPE65* (Tyr368His) was detected in one out of thirty-eight patients in a screening approach that included 103 mutations by an allele-specific ligation method (Sundaresan et al., 2009). Direct sequencing of candidate genes in series of unrelated RP patients, particularly those with ADRP and isolate RP, has revealed mutations in rhodopsin, pre-mRNA splicing factor 31 (*PRPF31*), retinitis pigmentosa 1 (*RP1*) and inosine monophosphate dehydrogenase 1 (*IMPDH1*) genes. These studies suggest a low frequency of mutations in these genes, of below 2% among the patients evaluated (Gandra et al., 2008).

Whole genome mapping studies have been reported on extended families with RP and related disorders. The *RP28* locus was mapped by conventional linkage analysis in a four-generation consanguineous family with ARRP from Karnataka in Southern India (seventeen members studied), to a region of about 1 cM on chromosome 2p11-p15 (Kumar et al., 2004). The age at onset of disease in this family was in the first-second decades of life, with features fairly typical of RP. Another genome wide linkage study mapped the disease on to chromosome 19q13.4 in a four-generation family with ADRP including eighteen members analyzed. A seven base pair deletion, leading to a predicted frameshift (p.Gly20AlafsX43), was identified in the *PRPF31* gene, which is present in the critical region. This family had incomplete penetrance of the disease (Saini et al., 2012). Clinical features in relation to the appearance of the fundus in the families reported in the two above-mentioned studies include pallor of the optic disk, attenuated retinal vasculature and bone spicule type of pigmentary deposits. Another family from North India was mapped to the same locus by linkage analysis at specific candidate gene loci (Bhatia et al., 2018). Patients in this family had a missense mutation of cysteine-299 to tyrosine (Cys299Tyr) in the *PRPF31* gene. Apart from the typical phenotypic features of RP as noted above, incomplete penetrance of the disease was again observed here. This phenomenon is a distinguishing aspect in families with *PRPF31* mutations (Vithana et al., 2003).

A whole genome linkage study led to the mapping of ADRP to chromosome 6q23, in a large four generation family from Andhra Pradesh (Kannabiran et al., 2012).

The analysis of autosomal recessive RP (ARRP) or other retinal dystrophy in Indian families has frequently employed homozygosity screening since the families studied are often consanguineous. The approach involves the detection of regions of homozygosity that are shared between affected offspring of a consanguineous marriage. The principle of mapping recessive disease genes in such inbred families relies on the presence of homozygosity by descent (HBD) in the genome of children of consanguineous parents (Lander and Botstein, 1987). Homozygosity mapping has been carried out with different types of genetic markers such as microsatellite markers and SNPs, and candidate genes in the regions of homozygosity thus detected, are screened for mutations. Often, a pedigree of a family with recessive disease is not suitable for detecting linkage due to its small size and limited number of affected offspring. Screening for homozygosity in such cases relies on detecting shared regions of homozygosity (ROH) at known disease loci among affected members of a family. More recently, the HBD principle has been applied to data from direct exome sequencing. In the latter technique, one looks at continuous stretches of shared homozygosity in the exome sequence of affected siblings to find the pathogenic mutations–in other words, it is an analysis of the autozygome.

Screening for HBD has led to successful identification of mutations in a subset of families with ARRP. Mapping with locus-specific microsatellite markers at candidate gene loci led to mutations being detectable in about 10%–15% of Indian families screened by this method (Singh et al., 2006; Singh et al., 2009). The genes associated to the disease include ATP-binding cassette subfamily A member 4 (*ABCA4*), tubby-like protein 1 (*TULP1*), retinaldehyde binding protein 1 (*RLBP1*), retinal pigment epithelial 65 kDa protein (*RPE65*) and retinitis pigmentosa 1 (*RP1*). Mutation in each gene listed here occurred in one family, and included frameshift, nonsense and missense changes. Screening for HBD with SNP arrays followed by sequencing of chosen genes, identified mutations in three out of sixteen families (one-fifth of families being positive for mutations) (Kannabiran et al., 2012). The onset of disease in the aforementioned ARRP families (Singh et al., 2009; Kannabiran et al., 2012) was in the 1st–2nd decades of life. Common features of the disease included arterial narrowing, disc pallor and diffuse degeneration of the RPE. Macular degeneration was present in some cases. Pigmentary deposits within the retina were not frequently observed. Another study from Southern India screened twelve families (eleven with LCA and one with ARRP) for homozygosity using SNP arrays and detected mutations in eleven families (Srilekha et al., 2015)- genes with pathogenic mutations in these patients were Aryl Hydrocarbon Interacting Protein-Like 1 (*AIPL1*), Retinal Pigment Epithelial 65 kDa Protein gene (*RPE65*), Guanylate Cyclase 2D (*GUCY2D*), Crumbs Homolog 1 (*CRB1*), Retinol Dehydrogenase 12 (*RDH12*), IQ Calmodulin-binding Motif-containing Protein 1 (*IQCB1*)*,* Spermatogenesis Associated Protein 7 (*SPATA7*) and Mer-Tyrosine Kinase (*MERTK*)*.* The associated phenotypes included typical changes in the fundus such as pigmentary deposits, atrophic macula, disc pallor and attenuated vasculature. A similar approach of homozygosity mapping with SNP arrays in three consanguineous families with different retinal dystrophies including LCA, ARRP and cone-rod dystrophy (CRD), was used to identify regions of homozygosity in the patients. This was followed by targeted NGS to identify the pathogenic changes in each family (Sundaramurthy et al., 2016). In this study, mutations were detected based on their location within one of the regions of homozygosity (ROH). Mutations in the *RDH12*, *ABCA4* and Cadherin-Related Family Member 1 (*CHDR1*) genes were found in the three families. Due to the presence of several large regions of homozygosity in the affected offspring in each family, the authors employed targeted NGS with a panel of 184 genes associated with eye diseases, to identify the relevant mutations. A related approach is that of whole genome homozygosity mapping in combination with exome sequencing. With this method, the disease was mapped to chromosome 14q31 in a large family, and whole exome sequencing showed a mutation in the tetratricopeptide repeat domain 8 (*TTC8*) gene in this locus (Goyal et al., 2016). The mutation was a missense change with substitution of glutamine-449 to histidine (p.Gln449His). Thus homozygosity mapping helped in the detection of pathogenic changes from the exome sequence data since one can restrict the variants that are selected to those present within the ROH.

Direct sequencing of candidate genes is a common approach employed for identifying mutations in all forms of RP. In particular, there are a large number of RP patients that are categorized as “isolate” or simplex RP. Candidate genes are selected on the basis of their known association with the disease in the same or other populations, or on their physiologic role and relevance in the functioning of photoreceptors or RPE. Screening of a chosen retinal disease gene has also been done on the basis of the associated phenotype. An example is a study of a four-generation family with two affected individuals and eleven members. Here, the affected members presented with clinical and electrophysiological findings suggestive of Goldman Favre syndrome (GFS). The retinal features suggestive of GFS included clumped pigment deposits in the fundus, with exudation and vascular masses in the peripheral retina (Manayath et al., 2014). Mutations in the *NR2E3* gene are known to be associated with GFS and clumped pigmentary retinal degeneration; analysis of this gene in the family detected a mutation of c.1117A > G leading to a missense change of aspartic acid-406 to glycine (Asp406Gly).

Targeted NGS is an extension of the candidate gene approach is, and is a multiplexed method for screening many genes in parallel for detecting mutations. Using this method, analysis of twenty candidate genes in ninety-two probands with LCA from southern India led to the detection of mutations in about 60% of cases (Srikrupa et al., 2018). A similar investigation involving 152 patients with RP in a tertiary care referral centre in southern India employed a targeted NGS panel of sixty-four genes. An unusually high frequency of mutations was obtained for the ceramide Kinase-Like 1 (*CERKL*) gene in this study. Mutations were found in fourteen patients, out of whom twelve unrelated patients had a common mutation of c.1045_1046delAT. One patient had a nonsense mutation of c.847C > T (arginine-257 to stop; Arg257Ter), and another had a splice site mutation of c.899-1 G > A. Characteristic features such as disc pallor, arteriolar attenuation, and variable degree of bony spicules were observed in these patients, along with macular atrophy (Sen et al., 2020).

Recent studies have entailed whole exome analysis (WES) by itself, in the absence of mapping. This mode has successfully found disease-associated mutations in RP in certain studies in which single families and even single patients were screened. An example is a study of two consanguineous families from southern India with two affected individuals each. A mutation in the *FAM161A* gene was detected by WES, comprising a common frameshift mutation at arginine-592 with termination after two amino acids (Arg592fsX2) (Zhou et al., 2015). Interestingly, in this study, screening of a series of 100 sporadic patients with RP identified one more unrelated patient with the same mutation. Another investigation with WES of two affected members in an Indian family with early-onset RP and loss of vision within the first decade, detected a splice site mutation (c.1160 +1G > A) in the Aryl Hydrocarbon Receptor (*AHR*) gene (Zhou et al., 2018). The mutation caused skipping of exon 9 of the *AHR* gene. Another consanguineous family from north India with eleven members with early-onset rod-cone dystrophy studied by WES, had a nonsense mutation (tyrosine-549 to Stop; c.1647T > G; Tyr549Ter) in the Mer-Tyrosine Kinase (*MERTK*) gene (Bhatia et al., 2019). Affected individuals in this family had pigment deposits, disc pallor, and attenuated retinal vessels in addition to diffuse changes in the RPE. Finally, WES analysis of a single child with RP from a consanguineous family in Andhra Pradesh detected a substitution mutation in the *TULP1* gene with change of proline-388 to serine (c.1162C > T; Pro388Ser). The patient presented with parafoveal atrophy of the RPE, bone spicule pigmentation in the mid-peripheral retina and attenuation of the retinal vasculature (Woodard et al., 2021). The mutation was observed to reduce the stability of the protein as compared to the wild type when expressed *in vitro*.

Apart from RP and LCA, there are fewer reported genetic studies of other retinal dystrophies in Indian patients. One such example is that of Best disease in families from southern India. Autosomal recessive bestrophinopathy (ARB) and Best vitelliform macular dystrophy (BVMD) are both associated with mutations in the Bestrophin 1 (*BEST1*) gene. BVMD is autosomal dominant in inheritance. Exome sequencing of four families, with a total of eight affected members and twelve unaffected members led to the detection of mutations in the *BEST1* gene in all cases (Nguyen et al., 2018). The patients presented with visual loss in the first to third decades of life and the changes observed in the fundus were foveal schisis, subretinal fluid and vitelliform deposits. The mutations detected in four separate families were-1) c.392A > G with a change of tyrosine 131 to cysteine (p.Tyr131Cys); 2) missense change of arginine-150 to proline (p.Arg150Pro); 3) mutations c.140G > A (arginine-47 to histidine; Arg47His) and c.646G > A (valine-216 to isoleucine; p.Val216Ile) in compound heterozygous patient; 4) threonine-91 to isoleucine (p.Thr91Ile) in a heterozygous proband. The first two mutations were found in homozygous patients.

In addition, autosomal recessive cone dystrophy in a single consanguineous family from northern India is associated with a mutation in the *CNGB3* (cyclic nucleotide gated channel subunit B3) gene (Gupta et al., 2017). The disease in this family manifested as poor vision since childhood, photophobia and abnormal color vision. The fundus showed vascular attenuation, with changes in the macular and mid-peripheral RPE. Electroretinographic responses of cones were abnormal. Exome sequencing led to the pathogenic change, a single base deletion of c.1148delC in *CNGB3* predicting frameshift at threonine-383 (p.Thr383Fs), with homozygosity in the affected individuals.

## 5 Genetics of Retinal Dystrophies in Pakistan

Analysis of pedigrees of extended families from Pakistan, often consanguineous, facilitated the identification of many new loci for retinal dystrophies. Table 2 has several examples of novel loci that were mapped using a range of strategies, depending on the technologies available. In many earlier studies, methods utilized to successfully find the disease gene were linkage analysis and sequencing of candidate genes in the mapped region. More recently, particularly over the last decade, exome sequencing has been the dominant method to find the genes involved. In some instances as in the discovery of the *USH1F* gene, comparison with a mouse deafness model for the disease, the *av* mouse, paved the way to finding the human gene (Ahmed et al., 2001). The disease gene in the *av* mouse, *Pcdh15*, was already known. Synteny between the human and mouse chromosome 10 suggested that the human orthologue of the mouse protocadherin 15 was the *USH1F* gene. Linkage analysis of two families with Usher syndrome refined the *USH1F* locus. Physical mapping of the critical interval for *USH1F* on chromosome 10q21.1, and detection of sequence similarity in this region to the murine *Pcdh15* gene led to identification of the human gene *PCDH15*.

Another locus on chromosome 6q, the *RP25* locus was also mapped through a combination of linkage and physical mapping. In this case, two separate studies mapped the locus on chromosome 6q. Homozygosity mapping in a subset of Spanish families with ARRP (Ruiz et al., 1998), and an independent study of a large consanguineous pedigree from Pakistan, comprising twenty individuals, both localized the gene to an interval of 2 cM on chromosome 6q (Khaliq et al., 1999). Further bioinformatic and transcript analysis of the sequences in the *RP25* genomic interval revealed a novel gene, *EYS* (human ortholog of the *drosophila* Eyes Shut gene). The coding regions of *EYS* included 44 exons that extended over 2 megabases of genomic DNA. Mutations were found in three unrelated families with RP in this gene, thus confirming its role in the pathogenesis of the disease (Collin et al., 2008).

The application of NGS has spurred the identification of genes that were mapped in earlier studies by linkage. An example is the *RP32* locus, mapped on to chromosome 1p13-21 (Zhang, Zulfiqar, Xiao et al., 2005), and identified by exome sequencing. Analysis of the sequence variants within the critical interval identified the *RP32* gene as the Chloride Channel CLIC like 1 (*CLCC1*) gene. An ancestral mutation was found in several families of Pakistani origin (Li et al., 2018). The *CLCC1* gene encodes a putative chloride channel and its knockdown in zebrafish and mouse has deleterious effects on the photoreceptor layer of the retina. Comparable to the above method is a combination of homozygosity mapping together with whole exome sequencing. With this strategy, analysis of a family of four affected individuals with ARRP using SNPs led to a single homozygous region being detected on chromosome 16. Analysis of exome variants located in the mapped interval resulted in identification of the pathogenic change in the pre-mRNA splicing factor gene, *DHX38* (Ajmal et al., 2014).

Several more studies on families with retinal dystrophy from Pakistan have detected mutations in known genes and they will not be detailed here. There is a paucity of research on retinal dystrophies from other south Asian countries including Nepal and Bangladesh and they appear to be truly understudied in this aspect with very few reports on these disorders, apart from occasional cases and prevalence studies of blindness that are hospital-based.

## 6 Conclusion

There is enormous scope for the investigation of the genetics of retinal dystrophies in India in particular and in South Asia, where the knowledge available in this field is very sparse in relation to the size of the affected population. The studies reported so far are indicative of the potential for gene discovery and for advancing our understanding of the genetic basis of these diseases in the region. The geographic and physical connectivity within families is still an inherent part of the social fabric. Such a situation offers an advantage to genetic studies of retinal dystrophies regardless of the methods that are employed, since the inclusion of extended families increases the scope for finding the underlying genes. Communities with a high degree of consanguinity and inbreeding may provide an opportunity to find genes that are more prevalent in them, due to common ancestry. This knowledge can eventually be used for targeted testing and counseling of families or for designing suitable therapies based on the genes involved.
